# *Parkia biglobosa* Improves Mitochondrial Functioning and Protects against Neurotoxic Agents in Rat Brain Hippocampal Slices

**DOI:** 10.1155/2014/326290

**Published:** 2014-08-10

**Authors:** Kayode Komolafe, Tolulope M. Olaleye, Rodrigo L. Seeger, Fabiano B. Carvalho, Aline A. Boligon, Margareth L. Athayde, Claudia V. Klimaczewski, Akintunde A. Akindahunsi, Joao B. T. Rocha

**Affiliations:** ^1^Departamento de Bioquímica e Biologia Molecular, CCNE, Universidade Federal de Santa Maria, 97105-900 Santa Maria, RS, Brazil; ^2^Department of Biochemistry, School of Sciences, Federal University of Technology, Akure 340001, Nigeria; ^3^Departamento de Farmácia Industrial, Universidade Federal de Santa Maria, 97105-900 Santa Maria, RS, Brazil

## Abstract

*Objective*. Methanolic leaf extracts of *Parkia biglobosa*, PBE, and one of its major polyphenolic constituents, catechin, were investigated for their protective effects against neurotoxicity induced by different agents on rat brain hippocampal slices and isolated mitochondria. *Methods*. Hippocampal slices were preincubated with PBE (25, 50, 100, or 200 *µ*g/mL) or catechin (1, 5, or 10 *µ*g/mL) for 30 min followed by further incubation with 300 *µ*M H_2_O_2_, 300 *µ*M SNP, or 200 *µ*M PbCl_2_ for 1 h. Effects of PBE and catechin on SNP- or CaCl_2_-induced brain mitochondrial ROS formation and mitochondrial membrane potential (ΔΨ*m*) were also determined. *Results*. PBE and catechin decreased basal ROS generation in slices and blunted the prooxidant effects of neurotoxicants on membrane lipid peroxidation and nonprotein thiol contents. PBE rescued hippocampal cellular viability from SNP damage and caused a significant boost in hippocampus Na^+^, K^+^-ATPase activity but with no effect on the acetylcholinesterase activity. Both PBE and catechin also mitigated SNP- or CaCl_2_-dependent mitochondrial ROS generation. Measurement by safranine fluorescence however showed that the mild depolarization of the ΔΨ*m* by PBE was independent of catechin. *Conclusion*. The results suggest that the neuroprotective effect of PBE is dependent on its constituent antioxidants and mild mitochondrial depolarization propensity.

## 1. Introduction

A significant body of scientific findings supports that excessive generation of ROS and the resulting oxidative stress play a significant role in neurodegenerative diseases [1–3]. Studies with neurodegenerative disease models have shown the involvement of nitric oxide elevation and mitochondrial dysfunction including the accompanying energy breakdown, intraneuronal calcium imbalance with increased expression of apoptotic proteins, and depletion of reduced glutathione levels in tissues [4–6]. Damage to brain cells in many neurodegenerative diseases usually arises from various physiological conditions and external insults that lead to increased free radical production which weakens the brain's antioxidants status and triggers the production of certain proteins and consequently promoting cellular death [7].

Mitochondrial electron transport chain is an important source of ROS generation in the form of superoxide anion radical (O_2_^•−^) in somatic cells [8]. The rate of O_2_^•−^ production has been reported to depend on the mitochondrial potential [9]. Of particular neurotoxicological significance, oxidative stress resulting from mitochondrial dysfunction is supposed to be a characteristic of many types of neurodegenerative diseases [10]. Mitochondrial dysfunction has deleterious consequence for cellular function and viability and, depending on the severity of the condition, can result in cellular death [10]. Mitochondria thus control the neuronal cell fate via mediation of apoptotic and necrotic cell death [11].

The acetylcholinesterase and Na^+^,  K^+^-ATPase enzymes are of considerable importance to neuronal functions. The latter is responsible for the consumption of about 50% of ATP generated in the brain cellular membrane which is utilized for the maintenance and reestablishing of the electrochemical gradients necessary for neuronal excitability and regulation of neuronal cell volume. The activity of the Na^+^,  K^+^-ATPase enzyme is known to decrease under certain pathophysiology conditions related to psychiatric disorders [12].

This whole set of knowledge suggests that compounds or natural products with beneficial effects on mitochondrial membrane function and antioxidant status might be of pharmacological usefulness in modulating or counteracting mitochondrial dysfunction in neurodegenerative diseases. In this regard, natural products or bioactive components with antioxidant and neuroprotective properties have been reported to exhibit preventive or therapeutic effects on experimental models of brain oxidative stress [13–16].

*Parkia biglobosa* (Jacq.) Benth., commonly known as “African locust bean,” is perennial deciduous tree with extensive uses in West Africa for food, medicine, and timber. The tree is known as* Igi iru* or* Irugba* among the Yoruba people of South-Western Nigeria where the seeds are fermented to make a strong smelling and tasty soup condiment rich in protein popularly called* Iru* [17]. It has popular ethnomedicinal use in tropical Africa in the treatment of hypertension and fevers [18–20] and as a major constituent of herbal preparations used as neurostimulant and in treatment of fatigue in South-Western Nigeria (oral communications by local herbal practitioners).* In vitro* antioxidant property of the crude ethanolic extract of the leaf and stem bark was reported [19]. We observed a potent antioxidant activity and beneficial effect of the methanolic leaf extract of the plant on isolated hepatic mitochondria [21] and recently reported the hypoglycemic effect of its constituent saponins mixture [22].

To the best of our knowledge, there is no data on the effect of* P. biglobosa* on brain mitochondrial integrity or its neuroprotective effect in the literature. The present study was therefore conducted to fill this lacuna by providing scientific information on the effect of* P. biglobosa* on enzymes of neurological significance, brain mitochondrial redox status, and hippocampal neuronal cell damage induced by different neurotoxicants in rats.

## 2. Materials and Methods

### 2.1. Chemicals

Ouabain octahydrate, adenosine triphosphate (ATP), acetylthiocholine iodide, 3(4,5-dimethylthiazol-2-yl)-2,5-diphenyltetrazolium bromide (MTT), dichlorofluorescein diacetate (DCFH-DA), 5,5^1^-dithiobis-(2-nitrobenzoic acid)(DTNB), catechin, (−) epigallocatechin, (−) epigallocatechin gallate, quercetin, rutin, and kaempferol were acquired from Sigma Chemical Co. (St. Louis, MO, USA). Dibasic phosphate potassium (K_2_HPO_4_), monobasic phosphate potassium (KH_2_PO_4_), and trichloroacetic acid (TCA) were supplied by Vetec (Rio de Janeiro, RJ, Brazil). All chemicals and solvents were of analytical grade and the water used was glass distilled.

### 2.2. Plant Material

Fresh leaves of* Parkia biglobosa* were collected in Isua-Akoko, Ondo State, Nigeria. Botanical identification and authentication were carried out by Dr. Ugbogu A. O. and Mr. Shasanya O. S. at the herbarium of the Forestry Research Institute (FRIN) Ibadan, Oyo State, Nigeria, where a voucher specimen (number 109603) was deposited.

### 2.3. *Parkia biglobosa* Extract Preparation

Air-dried leaves were ground to fine powder using a blender. A 500 g sample of the powdered material was macerated in 1200 mL of a mixture of methanol and water (4 : 1) for 48 hours. The filtrate obtained was concentrated to a small volume to remove the entire methanol using rotary evaporator. The concentrated extract was then lyophilized and kept at −20°C until required [23, 24]. Extract yield was approximately 11%. In each case, extract was reconstituted in water to give specific concentrations (in mg/mL or mg/mL) prior to use.

### 2.4. Quantification of Phenolics

High performance liquid chromatography (HPLC-DAD) was performed with a Shimadzu Prominence Auto Sampler (SIL-20A) HPLC system (Shimadzu, Kyoto, Japan), equipped with Shimadzu LC-20AT reciprocating pumps connected to a DGU 20A5 degasser with a CBM 20A integrator, SPD-M20A diode array detector, and LC solution 1.22 SP1 software. Reverse phase chromatography analyses were carried out under gradient conditions using a Phenomenex C-18 column (4.6 mm × 150 mm) packed with 5 *μ*m diameter particles. The mobile phase was water containing 2% acetic acid (A) and methanol (B), and the composition gradient was 5% of B until 2 min and was changed to obtain 25%, 40%, 50%, 60%, 70%, and 100% B at 10, 20, 30, 40, 50, and 60 min, respectively, following the method described by Sabir et al. [25] with slight modifications. Methanolic leaf extract of* Parkia biglobosa*, PBE, was analyzed after dissolution in methanol at a concentration of 10 mg/mL. The presence of nine compounds was investigated, namely, gallic acid, chlorogenic acid, caffeic acid, catechin, epigallocatechin, epigallocatechin gallate, quercetin, rutin, and kaempferol. Identification of these compounds was performed by comparing their retention time and UV absorption spectrum with those of the commercial standards. The flow rate was 0.8 mL/min, injection volume was 50 *μ*L, and the wavelength were 254 nm for gallic acid, 280 for catechin, epigallocatechin, and epigallocatechin gallate, 325 nm for chlorogenic and caffeic acids, and 365 nm for quercetin, rutin, and kaempferol. All the samples and mobile phase were filtered through 0.45 *μ*m membrane filter (Millipore) and then degassed by ultrasonic bath prior to use. Stock solutions of standards references were prepared in the HPLC mobile phase at a concentration range of 0.020–0.200 mg/mL for catechin, epigallocatechin, epigallocatechin gallate, quercetin, rutin. and kaempferol and 0.030–0.250 mg/mL for gallic, chlorogenic, and caffeic acids. The chromatography peaks were confirmed by comparing the retention time with those of reference standards and by DAD spectra (200 to 500 nm). All chromatography operations were carried out at ambient temperature and in triplicate. The limit of detection (LOD) and limit of quantification (LOQ) were calculated based on the standard deviation of the responses and the slope using three independent analytical curves, as defined by ICH [26]. LOD and LOQ were calculated as 3.3 and 10 *σ*/S, respectively, where σ is the standard deviation of the response and S is the slope of the calibration curve.

### 2.5. Animals

Male Wistar rats (±3 months old), weighing between 270 and 320 g, from the University breeding colony (Animal House Holding, UFSM, Brazil), were kept in cages with free access to foods and water in a room with controlled temperature (22°C ± 3) and in 12 h light/dark cycle with lights on at 7:00 a.m. The animals were maintained and used in accordance to the guidelines of the Brazilian association for laboratory animal science. All efforts were made to minimize animal suffering and to reduce the number of animals used.

### 2.6. Preparation of Brain Hippocampal Slices

Rats were decapitated, brains removed, and hippocampi dissected (4°C) in artificial cerebrospinal fluid (aCSF) containing (in mM) 120 NaCl, 0.5 KCl, 35 NaHCO_3_, 1.5 CaCl_2_, 1.3 MgCl_2_, 1.25 Na_2_HPO_4_, and 10 D-glucose (pH 7.4) [16]. Transverse sections (400 *μ*m) were prepared using a McIlwain tissue chopper (Campden instruments). The slices were preincubated at 37°C for 20 min before the start of each experiment.

### 2.7. Reactive Oxygen Species (ROS) Measurement

Fluorescent probes of 2′,7′-dichlorofluorescein diacetate (DCFH-DA) were employed in the determination of intracellular reactive species formation in hippocampal cells. The slices (4 slices per well) were incubated in aCSF in the presence of four concentrations of PBE (25, 50, 100, or 200 *μ*g/mL) or three concentrations of catechin (1, 5, or 10 *μ*g/mL) for 1 h and 30 min. Control slices in the present assay and others were treated with the appropriate volume of vehicle-distilled water. Slices of each sample were removed and homogenized in 1 mL of buffer before centrifuging at 3000 ×g for 10 min. To 200 *μ*L of the supernatant, 5 *μ*M DCFH-DA was added and ROS production in the slice homogenates was quantified by measuring the formation of the fluorescence product of DCFH oxidation (i.e., DCF). DCF fluorescence was monitored for 30 min at 2 min interval using excitation and emission wavelengths of 488 nm and 525 nm, respectively, in a microplate reader (SpectraMax M2e Multi-Mode, USA) as described by Wagner et al. [16]. Autooxidation of DCFH-DA was also measured in the absence of slices homogenate and the values were subtracted in each of the cases.

### 2.8. Assessment of Hippocampal Cellular Viability

**Figure 1:**
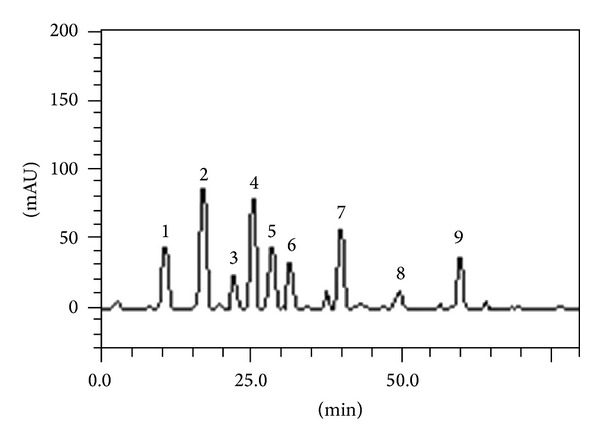
Representative high performance liquid chromatography profile of* Parkia biglobosa* leaf. ^a^Detection UV was at 325 nm. Gallic acid (peak 1), catechin (peak 2), chlorogenic acid (peak 3), caffeic acid (peak 4), epigallocatechin (peak 5), epigallocatechin gallate (peak 6), rutin (peak 7), quercetin (peak 8), and kaempferol (peak 9).

Cellular viability assay was performed by the colorimetric 3(4,5-dimethylthiazol-2-yl)-2,5-diphenyl tetrazolium bromide (MTT) method. MTT is widely used as an indicator of the mitochondrial activity of living cells which are capable of reducing the dye into a purple formazan product that is soluble in dimethyl sulfoxide (DMSO) [27]. The slices (4 slices per tube) were incubated in aCSF buffer in the presence of any of SNP (300 *μ*M), H_2_O_2_ (300 *μ*M), or PbCl_2_ (200 *μ*M) for 1 h and after pretreating in each case with four concentrations of PBE (25, 50, 100, or 200 *μ*g/mL) or three concentration of catechin (1, 5, or 10 *μ*g/mL) for 30 min. In another experiment, slices were treated with either PBE (25, 50, 100, or 200 *μ*g/mL) or catechin (1, 5, or 10 *μ*g/mL) alone to determine the effect of extract and its major flavonoid, catechin, on brain hippocampal cellular viability. 10 *μ*L of MTT (5 mg/mL, for a final concentration of 50 *μ*g/mL) was added and the plates were incubated for an additional 30 min at 37°C. Slices were removed and introduced into DMSO for complete extraction of the color. The optical density was measured using microplate reader (SpectraMax M2e Multi-Mode reader, USA) at 540 and 700 nm, and the net A540–A700 was taken as an index of cell viability [28]. Furthermore, the potential interference of the extract and some of the flavonoids (catechin, quercetin, and rutin) found in the extract (Figure 1) in the MTT reduction, without slices was investigated. The interference of these compounds and PBE was less than 1% of that observed in the presence of slices (data not shown). Thus, in 30 min of incubation, the chemical reduction of MTT by PBE or flavonoids was negligible.

### 2.9. Determination of Lipid Peroxidation in Hippocampal Slices

Measurement of lipid peroxidation was performed by detection of TBA-reactive substances as previously described [29] with slight modification. The slices (5 slices per tube) were incubated in an artificial cerebrospinal fluid (aCSF) in the presence or absence of any of SNP (300 *μ*M), H_2_O_2_ (300 *μ*M), or PbCl_2_ (200 *μ*M) for 1 h after preincubation in each case with four concentrations of PBE (25, 50, 100, or 200 *μ*g/mL) or three concentration of catechin (1, 5, or 10 *μ*g/mL) for 30 min. Hippocampal slices from each sample were then homogenized in 200 *μ*L of aCSF buffer. 50 *μ*L of homogenates was used for measurement of peroxidation by addition of 8.1% SDS (50 *μ*L), 1.35 M acetic acid buffer, pH 3.4 (120 *μ*L), and 0.8% TBA (80 *μ*L). The final solution was incubated at 100°C for 1 h. After cooling the samples on ice, they were centrifuged at 3,000 g for 10 min and the absorbance of the respective supernatants was measured spectrophotometrically at 532 nm using a microplate reader. The results were calculated as nmol of thiobarbituric reactive substances (TBARS) per mg of protein. The protein concentrations were determined by the method of Bradford [30] using bovine serum albumin (BSA) as standard.

### 2.10. Determination of Nonprotein Thiols (NPSH) Levels in Slices

Preincubation of hippocampal slices with PBE or catechin and treatment with toxicants proceeded as explained under lipid peroxidation determination. NPSH was determined by the method of Ellman [31] with slight modifications. Slices from each sample were homogenized in 10 mM Tris-HCl, pH 7.4. Briefly, a total of 100 *μ*L of tissue homogenates was precipitated with 50 *μ*L of 10% TCA (1 : 1 v/v) before subjecting to centrifugation at 3,000 ×g at 4°C for 10 min. Free –SH groups were determined in the protein-free clear supernatant (40 *μ*L) in an assay mixture containing 150 *μ*L of potassium phosphate buffer (1 M, pH 7.4) and 10 *μ*L DTNB (10 mM). The yellow color that developed was read at 412 nm in a microplate reader (SpectraMax M2e Multi-Mode, USA). The NPSH levels were calculated as *μ*mol NPSH/mg of protein.

### 2.11. Na^+^, K^+^-ATPase Activity Determination

The Na^+^,  K^+^-ATPase activity was measured in slices homogenate incubated with PBE or catechin as described by Wyse et al. [32] but with minor modifications. The assay medium consisted of (in mM) 30 Tris-HCl buffer (pH 7.4), 0.1 EDTA, 50 NaCl, 5 KCl, 6 MgCl_2_, and 50 *μ*g of protein in the presence or absence of ouabain (1 mM), in a final volume of 350 *μ*L. The reaction was started by the addition of adenosine triphosphate to a final concentration of 3 mM. After 30 min at 37°C, the reaction was stopped by the addition of 70 *μ*L of 50% (w/v) trichloroacetic acid. Appropriate controls were included in the assays for nonenzymatic hydrolysis of ATP. The amount of inorganic phosphate (Pi) released was quantified as previously described [33], using NaH_2_PO_4_ as reference standard. Specific Na^+^,  K^+^-ATPase activity was calculated by subtracting the ouabain-insensitive activity from the overall activity (in the absence of ouabain) and expressed in nmol of Pi/mg of protein/min.

### 2.12. Determination of Acetylcholinesterase Activity

AChE activity was measured by the slightly modified spectrophotometric method of Ellman et al. [34]. Briefly, in this method, 100 *μ*L of sodium phosphate buffer (100 mM, pH 7.5) containing 10 mM DTNB, 10 *μ*L of test solutions (PBE 25, 50, 100, and 200 *μ*g/mL or catechin 1, 5, and 10 *μ*g/mL concentrations) and 10 *μ*L of whole brain homogenate were added in a 96-well microplate and incubated for 5 min at 25°C. The reaction was then initiated with the addition of 20 *μ*L of acetylthiocholine iodide (8 mM). Hydrolysis of acetylthiocholine iodide was monitored by the formation of the yellow 5-thio-2-nitrobenzoate anion as a result of the reaction of DTNB with thiocholines catalysed by enzymes at 412 nm utilizing a 96-well microplate reader (SpectraMax M2e Multi-Mode reader, USA).

### 2.13. Isolation of Fresh Brain Mitochondria

Brain mitochondria were isolated as previously described by Brustovetsky and Dubinsky [35] with minor modifications. Wistar rats were killed by decapitation and the whole brain tissues were rapidly removed and placed on ice-cold isolation buffer containing 225 mM mannitol, 75 mM sucrose, 1 mM EGTA, 0.1% bovine serum albumin (BSA; free fatty acid), and 10 mM HEPES pH 7.2. The tissues were then homogenized and the resulting suspension centrifuged for 7 min at 2,000 ×g. Next, the supernatant was centrifuged for 10 min at 12,000 ×g. The pellet was resuspended in isolation buffer II containing 225 mM mannitol, 75 mM sucrose, 1 mM EGTA, and 10 mM HEPES pH 7.2 and centrifuged at 12,000 ×g for 10 min. Finally, the last supernatant was discarded, and the pellet was resuspended and maintained in buffer III (sucrose 100 mM, KCl 65 mM, K^+^-HEPES 10 mM, and EGTA 50 *μ*M pH 7.2) to a protein concentration of 0.5 mg/mL for subsequent analyses.

### 2.14. Evaluation of Reactive Species (RS) Formation with Dichlorofluorescein-Reactive Species (DCFH-RS)

RS levels were measured using the oxidant sensing fluorescent probe, 2′,7′-dichlorofluorescein diacetate (DCFH-DA) [36]. The oxidation of DCFH (which is formed by the action of esterase on DCFH-DA) to fluorescent dichlorofluorescein (DCF) was determined at 488 nm for excitation and 525 nm for emission. An aliquot of 5 *μ*L (50 *μ*g protein) of the homogenate of the isolated mitochondria was added to 3 mL of buffer III (containing 5 mM succinate). The reaction medium was exposed to PBE (25, 50, or 100 *μ*g/mL) or catechin (1, 5, or 10 *μ*g/mL) and/or 80 *μ*M Ca^2+^/150 *μ*M SNP. After 10 s, 10 *μ*M (DCFH-DA) (prepared in ethanol) was added to the mixture and the fluorescence intensity from DCF was measured for 300 s using a spectrofluorimeter (RF-5301 Shimadzu, Kyoto, Japan).

### 2.15. Measurement of Mitochondrial Membrane Potential (Δ*ψm*)

Mitochondrial membrane potential was estimated by fluorescence changes of safranin [37] recorded by a RF-5301 Shimadzu spectrofluorimeter (Kyoto, Japan) operating at excitation and emission wavelengths of 495 and 586 nm, respectively, with slit widths of 3 nm. Values of mitochondrial membrane potential (Δ*ψm*) were expressed relative to the control.

### 2.16. Statistical Analysis

Experiments conducted in replicates were expressed as mean ± standard error of the mean (SEM). Data on mitochondrial ROS production was analyzed using two-way analysis of variance followed by Bonferroni posttest to account for the two variables of time and concentration. Unless otherwise stated, other data were analyzed using one-way analysis of variance followed by the Newman-Keuls post hoc test.

## 3. Results

### 3.1. HPLC Analysis

HPLC analysis revealed the presence of flavonoids (catechin, epigallocatechin, epigallocatechin gallate quercetin, rutin, and kaempferol), and phenolic acids (gallic, chlorogenic, and caffeic acids) in the methanolic extract of* Parkia biglobosa *leaf (PBE). The chromatogram revealed the presence of gallic acid (retention time, *t*_*R*_ = 11.78 min; 1.53%; peak 1), catechin (*t*_*R*_ = 17.08 min; 2.94%; peak 2), chlorogenic acid (*t*_*R*_ = 22.97 min; 0.64%; peak 3), caffeic acid (*t*_*R*_ = 25.36 min; 2.81%; peak 4), epigallocatechin (*t*_*R*_ = 28.67 min; 1.50%; peak 5), epigallocatechin gallate (*t*_*R*_ = 32.05 min; 1.12%; peak 6), rutin (*t*_*R*_ = 39.83 min; 1.75%; peak 7), quercetin (*t*_*R*_ = 48.54 min; 0.41%; peak 8), and kaempferol (*t*_*R*_ = 60.15 min; 1.27%; peak 9) (Figure 1 and Table 1).

The results were similar to that obtained earlier on the same extract analyzed after dissolving in ethanol (unpublished data). The present analysis however made use of more standards for comparison.

### 3.2. Attenuation of Intracellular ROS Accumulation by PBE and Catechin

**Figure 2:**
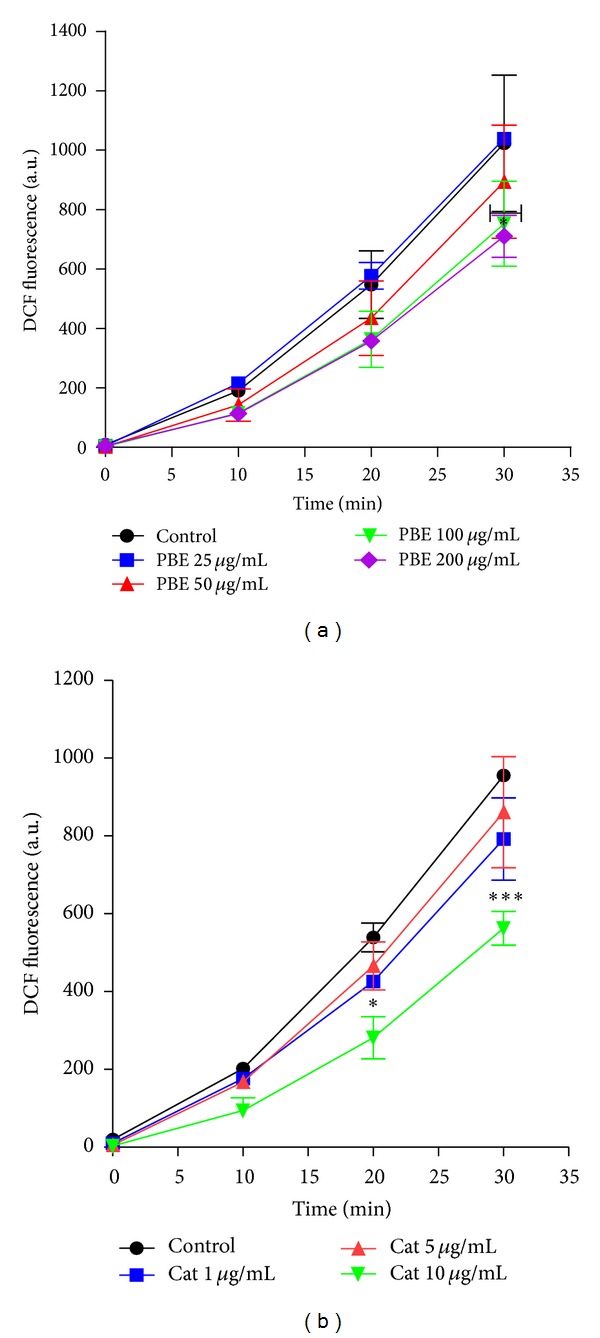
Effect of PBE (a) and catechin (b) on basal ROS generation in rats' brain hippocampal slices. Hippocampal slices were incubated in the presence or absence of* Parkia biglobosa* extract, PBE (25, 50, 100, or 200 *μ*g/mL), or catechin (1, 5, or 10 *μ*g/mL). Slices of each sample were then homogenized to obtain the supernatant in which ROS production was quantified by reacting with 5 *μ*M DCFH-DA. Data of ROS levels are presented as fluorescence intensity emission and expressed as mean ± SEM of three independent assays. Data analysis was done by two-way ANOVA, followed by Bonferroni posttests (*P* < 0.05 was considered statistically significant). Statistically significant mitigation of basal ROS generation was achieved at 100 and 200 *μ*g/mL concentration of PBE (a) and 10 *μ*g/mL concentration of catechin (b). *P < 0.05 and ***P < 0.001 versus untreated slices (control).

As shown in Figure 2, PBE caused dose-dependent decrease in basal ROS formation in hippocampal slices with the decrease becoming statistically significant at 100 and 200 *μ*g/mL concentrations. Catechin caused significant reduction in DCFH oxidation at 10 *μ*g/mL concentration.

### 3.3. Improvement of Cellular Viability/Mitochondrial Function by PBE and Catechin

**Figure 3:**
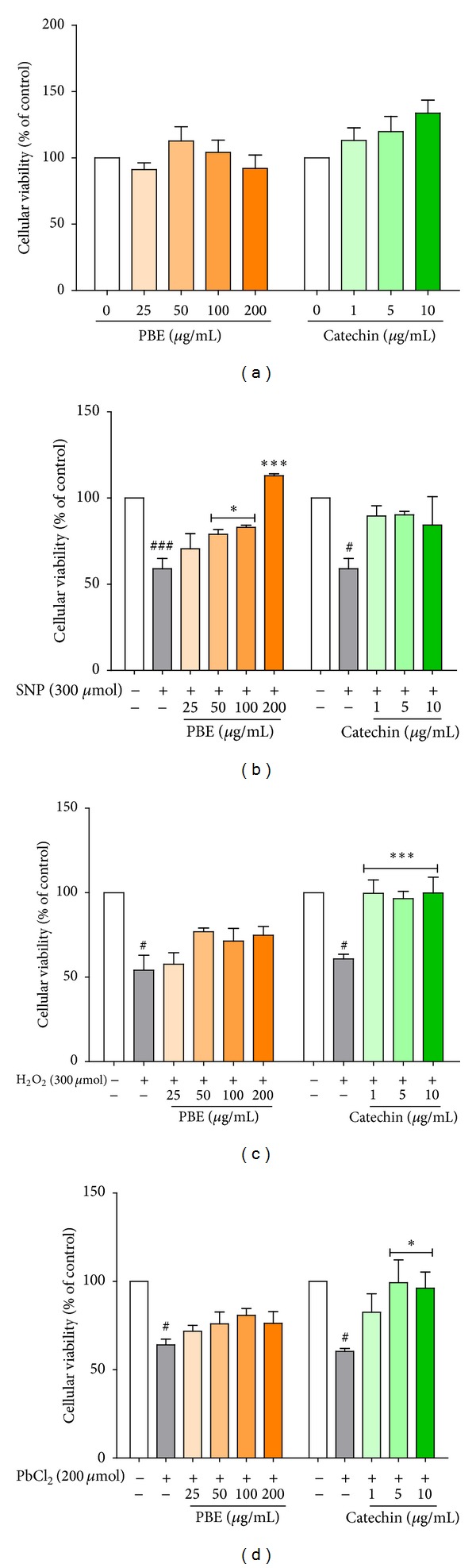
Modulation of cellular viability by PBE and catechin. Hippocampal brain slices were incubated with either* P. biglobosa* extract, PBE (25, 50, 100, or 200 *μ*g/mL), or catechin (1, 5, or 10 *μ*g/mL) alone for 1 h 30 min or preincubated with the same for 30 min before final incubation with any of the toxicants (SNP, H_2_O_2_, or PbCl_2_) for 1 h. The net color intensity of the purple formazan product formed following MTT staining and measured at 540 nm and 700 nm wavelengths (A540–A700) was taken as index of cellular viability. Data are expressed as mean ± SEM of three independent experiments.^ #^ *P* < 0.05, ^##^ *P* < 0.01, and ^###^ *P* < 0.001 versus untreated slices (control) and *P < 0.05, **P < 0.01, and ***P < 0.001 versus slices treated with toxicants (SNP, H_2_O_2_, or PbCl_2_) alone, as determined by one-way ANOVA followed by the Newman-Keuls multiple comparison test.

One-way ANOVA followed by the Newman-Keuls multiple comparison test revealed that treatment of hippocampal slices with PBE or catechin alone had no statistically significant effect on hippocampal cellular viability/mitochondrial function at the tested concentrations. A significant loss of cellular viability in SNP-treated slices was however observed. Pretreatment with PBE protected hippocampal slices from SNP-induced mitochondrial damage assessed by MTT reduction in a dose-dependent manner but the improvement by catechin was not significant (Figure 3(b)). H_2_O_2_-dependent decrease in viable hippocampal cells was blunted in catechin pretreated slices but the inhibition of H_2_O_2_ effect on slices by PBE was not statistically significant at any of the concentrations used (Figure 3(c)). Pretreatment with 5 or 10 *μ*g/mL concentration of catechin had beneficial effect on PbCl_2_ toxicity as it prevented PbCl_2_-dependent loss of hippocampal cells viability (Figure 3(d)).

### 3.4. Attenuation of Hippocampal Cells Membrane Lipid Peroxidation

**Figure 4:**
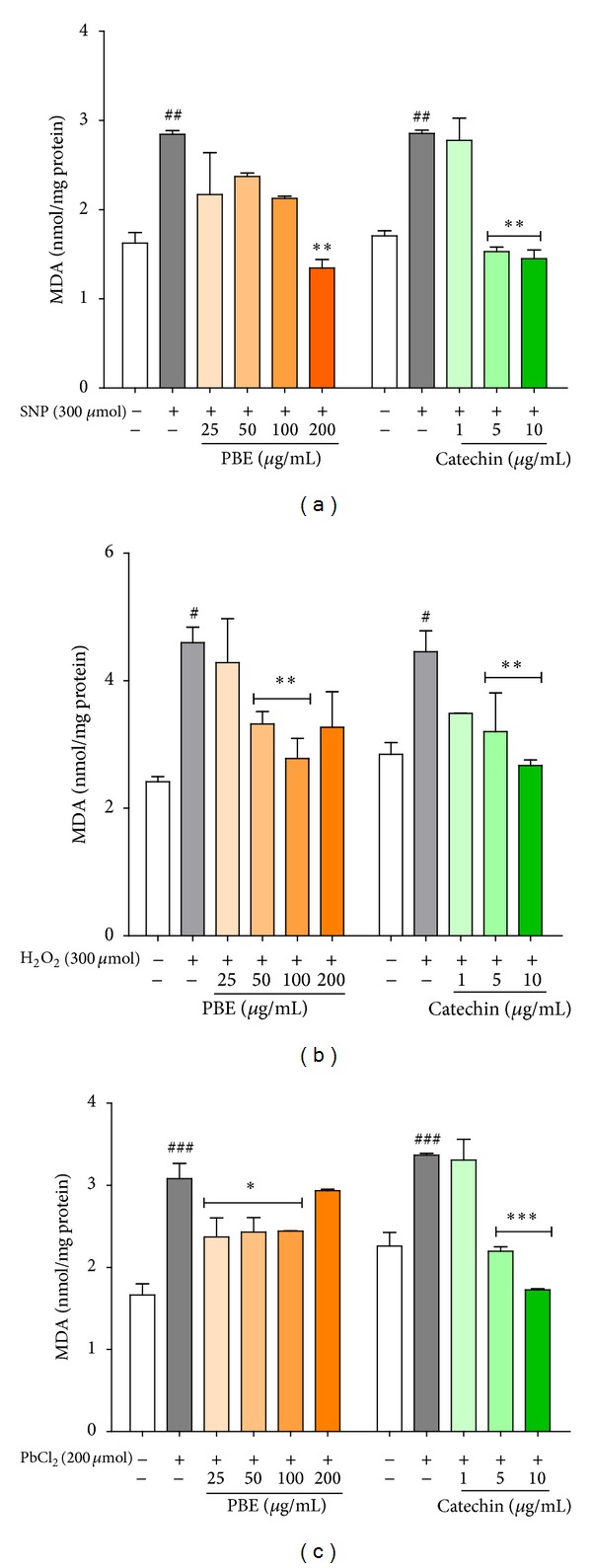
Attenuation of membrane lipid peroxidation in hippocampal cells by PBE and catechin. Hippocampal brain slices were preincubated with* P. biglobosa* extract, PBE (25, 50, 100, or 200 *μ*g/mL), or catechin (1, 5, or 10 *μ*g/mL) for 30 min before final incubation with toxicants (SNP, H_2_O_2_, or PbCl_2_) for 1 h. Thiobarbituric acid reactive species (TBARs) were quantified in slices homogenate and used as index of membrane lipid peroxidation. Data are expressed as mean ± SEM of three independent experiments. ^#^ *P* < 0.05, ^##^ *P* < 0.01, and ^###^ *P* < 0.001 versus untreated slices (control) and *P < 0.05, **P < 0.01, and ***P < 0.001 versus slices treated with toxicants (SNP, H_2_O_2_, or PbCl_2_) alone, as determined by one-way ANOVA followed by the Newman-Keuls multiple comparison test.

The toxicity of SNP (300 *μ*M) to the hippocampal cells was manifested by the increased rate of membrane lipid peroxidation. Statistically significant decrease in toxicant-induced peroxidation of hippocampal cells membranes was achieved by 200 *μ*g/mL PBE (Figure 4(a); *P* < 0.01) or 5 and 10 *μ*g/mL catechin (Figure 4(a); *P* < 0.001). Attenuation of H_2_O_2_-induced increase in hippocampal cells TBARs generation was evident in 50 and 100 *μ*g/mL PBE pretreated (Figure 4(b); *P* < 0.05) or 5 and 10 *μ*g/mL catechin pretreated (Figure 4(b); *P* < 0.01) hippocampal slices. As shown in Figure 4(c), similar protective effect against PbCl_2_-dependent damage was observed for slices pretreated with both PBE and catechin, respectively.

### 3.5. Effect of PBE and Catechin on Nonprotein Thiol (NPSH) Contents

**Figure 5:**
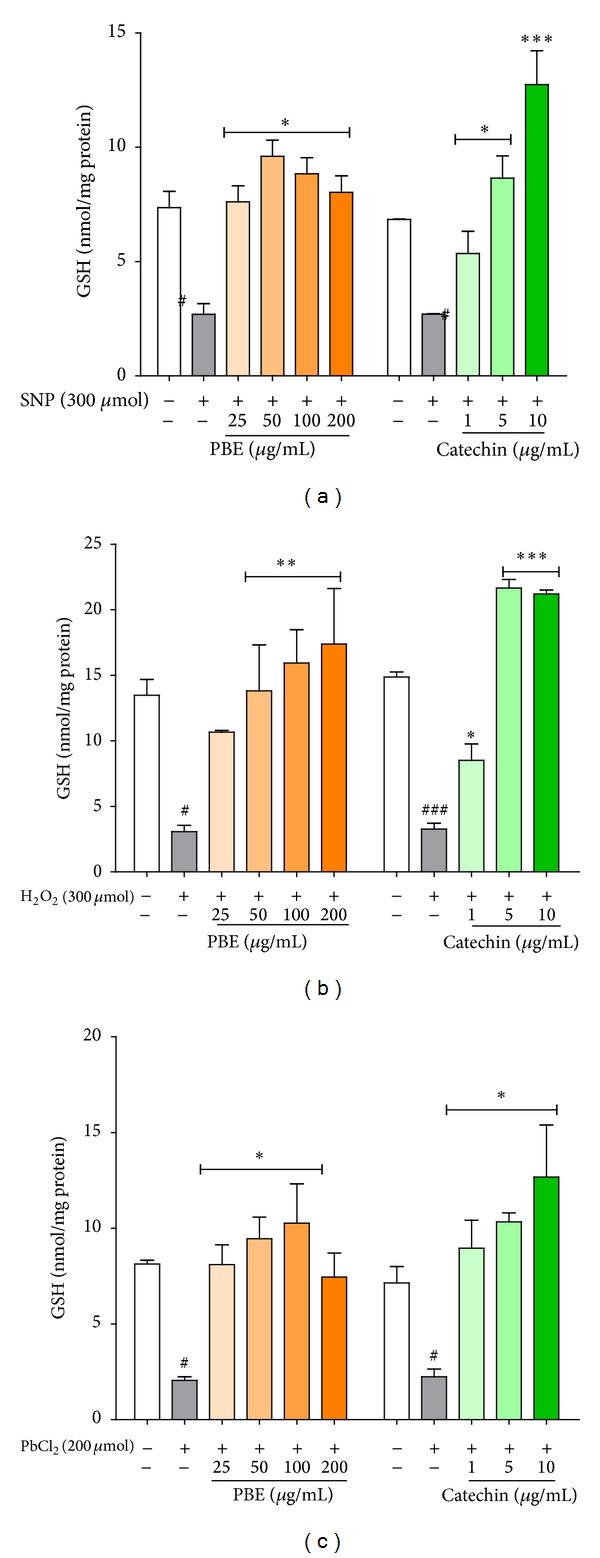
Effect of PBE and catechin on nonprotein thiol content. Hippocampal brain slices were preincubated with* P. biglobosa* extract, PBE (25, 50, 100, or 200 *μ*g/mL), or catechin (1, 5, or 10 *μ*g/mL) for 30 min before final incubation with toxicants (SNP, H_2_O_2_, or PbCl_2_) for 1 h. Slices homogenates were deproteinized and the free –SH groups were quantified in the protein-free supernatant by measuring the intensity of the color produced on reacting with Ellman's reagent at 412 nm. Data are expressed as mean ± SEM of three independent experiments. ^#^ *P* < 0.05 and ^##^ *P* < 0.01 versus untreated slices (control) and *P < 0.05 and ***P < 0.001 versus slices treated with toxicants (SNP, H_2_O_2_, or PbCl_2_) alone, as determined by one-way ANOVA followed by the Newman-Keuls multiple comparison test.

Reduced glutathione (GSH) comprises the bulk of cellular nonprotein thiol groups (NPSH). NPSH was significantly decreased in SNP (300 *μ*M), H_2_O_2_ (300 *μ*M), and PbCl_2_ (200 *μ*M) treated slices that were without prior pretreatment. PBE (50, 100, and 200 *μ*g/mL) pretreated slices showed increased NPSH levels in both cases of toxicant induction such that no significant difference could be observed when compared with normal, untreated slices (control). As can be observed in Figures 5(a), 5(b), and 5(c) also, similar boost in cellular nonprotein thiol contents was shown by catechin pretreated slices. Apparently, both PBE and catechin are involved in mechanisms capable of boosting cellular thiol contents under conditions of oxidative stress.

### 3.6. Effect of PBE on Na^+^, K^+^-ATPase Activity

**Figure 6:**
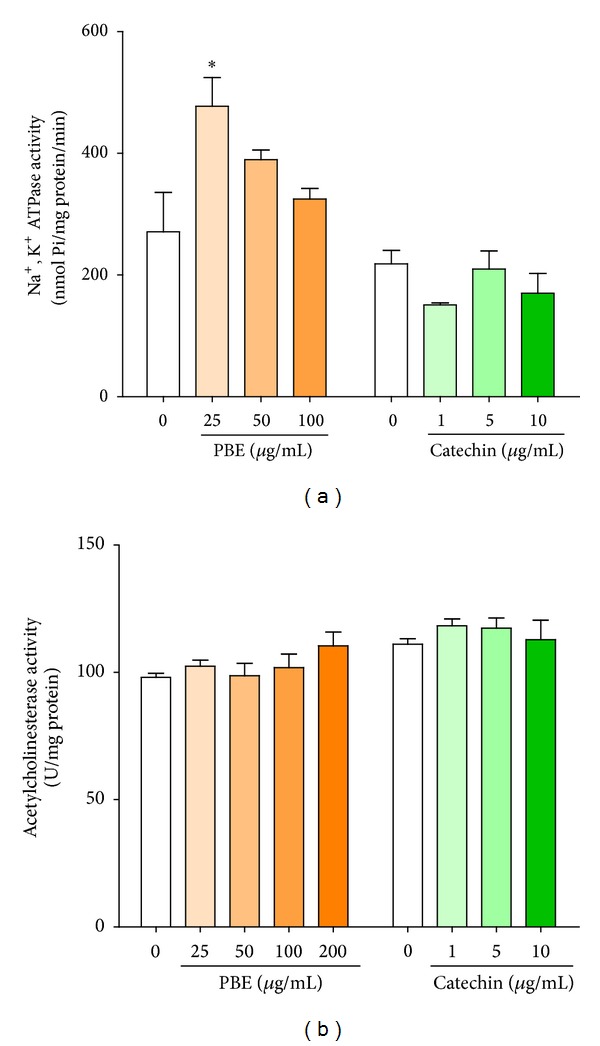
Effect of PBE and catechin on cerebral enzymes activities. Effect of PBE and catechin on Na^+^,  K^+^-ATPase (a) and total acetylcholinesterase activities (b) in rats' brain hippocampal slices homogenate. Hippocampal slices were preincubated with* P. biglobosa* extract, PBE (25, 50, 100, or 200 *μ*g/mL), or catechin (1, 5, or 10 *μ*g/mL) for 1 h and 30 min. Slices homogenates were assayed for the activity of Na^+^,  K^+^-ATPase. Data are expressed as mean ± SEM of three independent experiments. *P < 0.05 versus untreated slices/homogenate (control), as determined by one-way ANOVA followed by the Newman-Keuls multiple comparison test.

Na^+^,  K^+^-ATPase activity was significantly increased in PBE-treated slices (Figure 6(a); *P* < 0.05) but no effect was observed for catechin-treated slices. Both PBE and catechin however did not affect cerebral acetylcholinesterase activity at the evaluated concentrations.

### 3.7. PBE and Catechin Decrease Mitochondrial ROS Production

**Figure 7:**
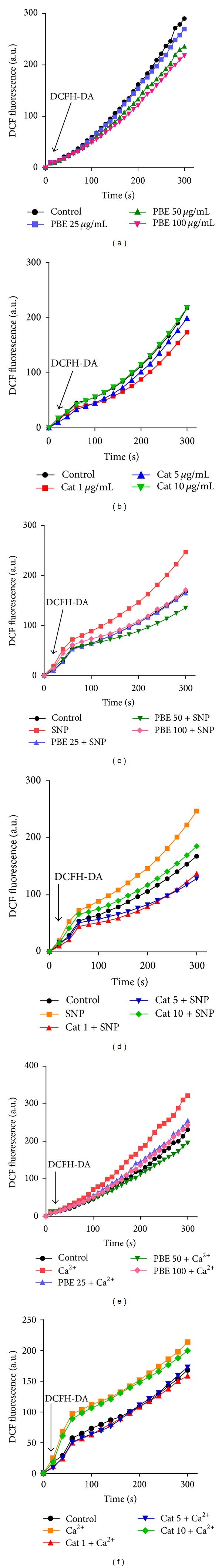
Effects of PBE and catechin on brain mitochondrial ROS production: SNP and Ca^2+^ interactions. Mitochondria were incubated in a medium containing 50 *μ*M EGTA, 10 mM sucrose, 65 mM KCl, 5 mM glutamate, 5 mM succinate, and 10 mM HEPES, pH = 7.2, and PBE (25, 50, or 100 *μ*g/mL) or catechin (1, 5, or 10 *μ*g/mL) in the presence or absence of 150 *μ*M SNP or 80 *μ*M CaCl_2_ for 10 s_._ The reaction was initiated by the addition of 2′,7′-dichlorofluorescein diacetate (DCFH-DA) and the fluorescence intensity emission arising from the oxidized fluorescent derivative (DCF) was measured over a 300 s period. Results are presented as mean ± SEM of three independent experiments. Data analysis was done by two-way ANOVA, followed by Bonferroni posttests (*P* < 0.05 was considered statistically significant). Indicators of statistical significance were not shown in the graphs above to avoid ambiguity.

Figures 7(a) and 7(b) show the effect of PBE and catechin on basal ROS formation in the mitochondria. Statistical analysis revealed that PBE reduced mitochondrial oxidative stress at 50 and 100 *μ*g/mL concentrations (*P* < 0.001) but the same feat was achieved by catechin only at 1 *μ*g/mL concentration.

Figures 7(c) and 7(d) show the effect of* Parkia biglobosa* leaf extract (PBE) and catechin on brain mitochondrial ROS production: interaction with SNP. Two-way ANOVA of data from Figures 7(c) and 7(d)yielded a significant main effect of SNP induction and a significant interaction between this factor and* P. biglobosa *and catechin concentrations, respectively. As can be observed, SNP caused an increase in DCFH oxidation, whereas all concentrations of PBE and two concentrations of catechin (1 and 5 *μ*g/mL) blunted the increase in oxidative stress production.

Figures 7(e) and 7(f) show the effect of* Parkia biglobosa* leaf extract (PBE) and catechin, respectively, on brain mitochondrial ROS production and the interaction with Ca^2+^. Similar to the observation under SNP induction, all concentrations of PBE and two concentrations of catechin (1 and 5 *μ*g/mL) inhibited the Ca^2+^ increase in DCFH oxidation to DCF.

### 3.8. PBE Exhibits Mild Mitochondrial Depolarization Potential

**Figure 8:**
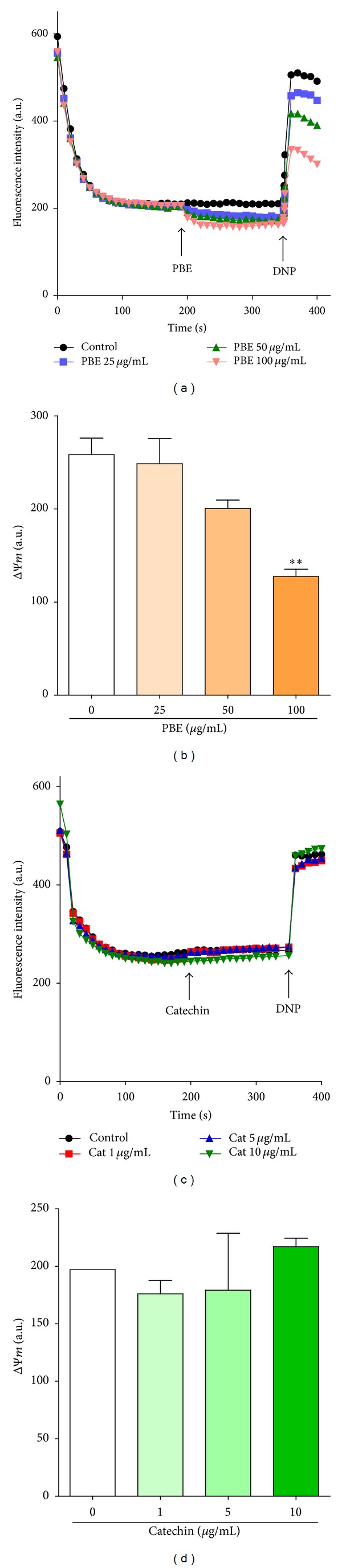
Effect of* Parkia biglobosa* leaf extract (PBE) (a) and catechin (c) on isolated rat brain mitochondrial potential and the respective potential difference (ΔΨ*m*) (b) and (d).

As shown in Figure 8(b), PBE caused dose-dependent but mild depolarization of the brain mitochondrial potential (ΔΨ*m*) which only becomes statistically significant at the highest concentration of 100 *μ*g/mL (*P* < 0.01) whereas catechin at the evaluated concentrations showed no effect on the brain mitochondrial potential (Figure 8(d)).

## 4. Discussion

Plants and plant-products will continue to find relevance in the treatment and management of numerous diseases and/or pathological conditions especially in the low-income countries due to their ready accessibility and inexpensive nature. The relatively safer and nontoxic nature of these natural products is also believed to give them an edge over their synthetic counterparts. HPLC analysis of the phenolics in the leaf of* P. biglobosa* revealed that the flavan-3-ols, catechin, epicatechin, and epigallocatechin, represent considerable portion of phenolics in the leaf with catechin being the most abundant. Catechin was therefore chosen as the reference phenolic in the present study and the concentration range investigated was based on the empirical evidence presented by the HPLC results. Catechin polyphenols in green tea were identified to be paramount to the neuroprotective properties of the green tea plant majorly through their strong antioxidant, free radical scavenging, and metal ion chelating activities [38]. They could also exhibit beneficial effects on the mitochondrial redox status [39, 40].

In the present study, we have investigated the effect of a leaf extract of a medicinal plant,* P. biglobosa*, and catechin on key enzymes of neurological significance and brain hippocampal slices toxicity induced by different neurotoxicants. Additionally, their effects on isolated brain mitochondrial ROS production and membrane potential were also investigated in realization of the involvement of these mitochondria parameters in neurotoxicity and in the pathology of many neurodegenerative diseases [10, 11]. The choice of hippocampus is based on the observation that this part of the brain is particularly sensitive to stress effects [41]. In this study, we sought to establish the relationship between the purported neuroprotectivity of* P. biglobosa* and the constituent phenolics and shed some lights on the underlying mechanism(s).

We observed a reduction in basal ROS generation in hippocampal slices with both PBE and catechin although the effect of catechin on basal ROS generation in the mitochondria was only significant at the lowest concentration. Mitigation of ROS generation by both PBE and catechin might be responsible for their ability to protect against the loss of hippocampal cellular viability in the presence of neurotoxicants. In consistence with Bastianetto and Quirion [42], exposure of rat hippocampal cells to SNP could have decreased cellular survival through increased mitochondrial production of reactive oxygen species (ROS). The catechin polyphenol and some other polyphenolics were capable of protecting hippocampal cells against SNP-induced toxicity and the neuroprotection might be more related to their antioxidant properties without involving the intracellular enzymes such as the NO synthase [43]. Mitigation of H_2_O_2_-induced hippocampal membrane peroxidation and prevention of the depletion of nonprotein thiol contents by PBE can also be related to the antioxidant phenolic constituents of which catechin is principal. The reactive oxygen species, H_2_O_2_, are produced in large quantities during redox processes and are capable of inducing membrane lipid peroxidation, DNA damage, and eventually leading to apoptosis in different cell types [44]. Therapeutic strategies aimed at preventing ROS effects including cellular apoptosis hold promises for the treatments of the numerous diseases in which excessive ROS generation is involved in their etiology [45]. Exposure to neurotoxic metals like lead (Pb) still occurs at relatively higher and toxicologically significant levels in the developing world especially in the urban environments of some Asian and African countries [46]. Pb^2+^ can cause learning and memory impairment at the developmental stages and detrimental effects at blood levels as low as 5 *μ*g/dL have been reported in children [47]. It is claimed that Pb exerts its toxicity via its inhibitory effect on the N-methyl-D-aspartate receptors in the hippocampus. Chronic exposure to low dose of the metal was shown to cause reduction in Ca^2+^-dependent glutamate and *γ*-aminobutyric acid (GABA) release in the hippocampus and overall presynaptic neuron dysfunction in rats [48, 49]. The freely diffusible and stable ROS, H_2_O_2_, could induce oxidative stress by promoting calcium influx and interacting with iron or copper to generate toxic ROS, including the highly potent hydroxyl radical which can result in neuronal cell death [50, 51]. H_2_O_2_ has been postulated to be involved in neurodegenerative disease and the neuronal injury and death induced by amyloid beta protein and glutamate [51]. Excessive calcium and lead could facilitate the generation of reactive oxygen species in biological systems. Oxidative stress-mediated cellular damage and loss of cellular viability may be involved in some of the pathologies associated with lead toxicity [52]. Elevated blood-lead concentration has been correlated with reduced level of antioxidant molecules like *α*-tocopherol and ascorbic acid in human [53]. Since both lead and H_2_O_2_ display oxidative stress-dependent approach to their toxicity, it is only logical to assume that antioxidants could be a vital component of an effective treatment. The catechins are strong scavengers of reactive oxygen and nitric radicals as well as effective metal chelators owing to their catechol structures [54]. The observed improvement of the viability of H_2_O_2_- and PbCl_2_-treated hippocampal cells by catechin in the present study could therefore be attributed, at least in part, to the antioxidant property of the polyphenol. We recently suggested that antioxidant activity and beneficial control of metal ion homeostasis in the cerebral system could be important neuroprotective strategies by some medicinal plants or other agents against neurodegenerative diseases like Alzheimer's and Parkinson's [55].

In the present study, whereas catechin prevented both the increased lipid peroxidation and reduced viability of hippocampal cells exposed to lead, PBE inhibited membrane peroxidation and increased thiol contents only. Elevation of membrane lipid peroxidation products and depletion of GSH levels in cells depict oxidative stress condition which could result in loss of cellular viability. The observation in the present study, however, that attenuation of oxidative stress condition in hippocampal cells by both PBE and catechin did not always translate to significant improvement in cellular viability however could be as a result of the relatively short incubation time with the extract or its major polyphenol.

Dysregulation of calcium signaling and the consequent generation of reactive oxygen and nitrogen species including nitric oxide (NO) have been implicated in mitochondrial dysfunction and of great significance in neurodegenerative disease [3]. In neuronal mitochondria, overproduction of NO make it readily available to react rapidly with the superoxide radical (O_2_^•−^) forming the very toxic peroxynitrite (ONOO–) and the consequent oxidative stress-mediated damage. In this regard, a possible pharmacological strategy to neurodegenerative diseases was suggested to be the prevention of Ca^2+^ mediated nitrosative stress [3]. The nitric oxide (NO) donor, sodium nitroprusside (SNP), has been reported to exhibit deleterious effect on mitochondrial function and has been capable of inhibiting the activity of complex IV of the mitochondrial electron transport chain (MTC) with consequent apoptotic cell death [56]. In the present study, both PBE at all concentrations used and catechin were effective in blunting SNP and Ca^2+^-dependent ROS generation in the mitochondria. In line with a previous finding [57] we hypothesize a direct scavenging of nitric oxide (NO) and superoxide radical (O_2_^•−^) by PBE through its phenolic constituents like catechin and other flavan-3-ols even though other mechanisms are not ruled out. We recently reported the protection against liver mitochondria damage by a plant extract through one or more of its antioxidant phytochemicals via antioxidant and Fe (II) chelating mechanisms [58].

Given the effect of both PBE and catechin on the ΔΨ*m*, the propensity of PBE for mild depolarization of the brain mitochondrial membrane potential could not be dependent on the catechin polyphenols but probably on one or more other phytoconstituents or the interactions among these. The neuroprotective effect of a plant extract was once attributed to the mild depolarization of ΔΨ*m* [59]. The mitochondrial membrane potential (ΔΨ*m*) contributes significantly to the extent of ROS production and with increased production occurring at high potentials [60]. The effect of PBE on ΔΨ*m* might be responsible for its superior efficacy in attenuating mitochondrial ROS formation compared to catechin alone. “Mild uncoupling” of mitochondria is believed to be turned on* in vivo* to diminish the formation of ROS [61] even though the mechanisms involved in this regulation of ΔΨ*m* are complex and not fully understood. Such decrease in the mitochondrial membrane potential (ΔΨ*m*) primarily attenuates mitochondrial ROS production with consequential decrease in mitochondrial Ca^2+^ uptake [62], preventing mitochondrial calcium overload and the subsequent apoptosis [63] of the neurons.

To provide further insight into mechanisms of PBE neuroprotectivity, we investigated the effect on cerebral acetylcholinesterase and Na^+^,  K^+^-ATPase activity which are both enzymes of neurological significance. Neither PBE nor catechin at the study concentrations possesses any inhibitory effect on the acetylcholinesterase enzyme but the result of PBE treatment revealed that the plant extract could boost the activity of Na^+^/ K^+^-ATPase in the cerebral tissue. Since Na^+^,  K^+^-ATPase is essential to brain normal function, modulation of this enzyme might contribute to the therapeutic efficacy and the neuroprotective effects of* Parkia biglobosa*. Some cases of psychiatric disorders are known to involve disruption in ion homeostasis and are often characterized by decreased Na^+^,  K^+^-ATPase activity [12, 64]. Decreased Na^+^,  K^+^-ATPase activity in the hippocampus of animals submitted to chronic mild stress was also reported [65]. By implication, drugs or natural products capable of augmenting the activity of this enzyme in such instances may be of immense benefit in ensuring the maintenance of the critical electrochemical gradient necessary for neuronal functions and hence neuronal viability.

Notwithstanding the observed effect of PBE on mitochondrial redox status and hippocampal cells viability, especially considering that the extract is a complex mixture of phytochemicals, it is impossible to extrapolate the findings from this* in vitro* study to* in vivo* situation.* In vivo* studies are still necessary to define the concentrations that could exert biological effects. However, considering the beneficial effects of catechin and other polyphenol constituents of PBE and the literature points of evidence, indicating the absorption of these components in mammals, we can suggest that sufficient absorption could occur to reach biologically relevant concentrations in the bloodstream [66, 67].

## 5. Conclusion

In summary, the results presented here suggest that PBE did exhibit neuroprotectivity by attenuating toxicant-induced ROS and oxidative stress in cerebral mitochondria and hippocampal tissue and boosting Na^+^,  K^+^-ATPase activity. The neuroprotective effect of* P. biglobosa* might be partly related to the antioxidant phenolics as well as its mild mitochondrial depolarization propensity by yet to be identified phytoconstituent(s) and in yet to be clarified mechanism(s). However, the results presented here cannot be extrapolated to* in vivo* situations, which is a limitation of the present study. Consequently,* in vivo* studies are needed to make appropriate assumptions about the safety and effectiveness of this plant extract as neuroprotective agent in mammals.

## Figures and Tables

**Table 1:** Phenolic and flavonoid compositions of methanolic leaf extract of *Parkia biglobosa*.

Compounds	*Parkia biglobosa *		LOD	LOQ
	mg/g	Percent	*μ*g/mL	*μ*g/mL
Gallic acid	15.38 ± 0.01	1.53	0.017	0.056
Catechin	29.42 ± 0.02	2.94	0.044	0.145
Chlorogenic acid	6.43 ± 0.05	0.64	0.036	0.119
Caffeic acid	28.19 ± 0.01	2.81	0.009	0.028
Epigallocatechin	15.04 ± 0.03	1.50	0.007	0.023
Epigallocatechin gallate	11.25 ± 0.02	1.12	0.016	0.054
Rutin	17.52 ± 0.01	1.75	0.022	0.074
Quercetin	4.16 ± 0.01	0.41	0.028	0.092
Kaempferol	12.79 ± 0.03	1.27	0.031	0.103

Results are expressed as mean ± standard deviations (SD) of three determinations.
